# Structure and Magnetic Properties of CNT-Reinforced Iron Composites

**DOI:** 10.3390/ma18194600

**Published:** 2025-10-03

**Authors:** Chunxia Zhou, Liang Yan, Biao Yan, Zhiya Han, Yixiao Cao, Xinyi Xu

**Affiliations:** 1 School of Materials, Shanghai Dianji University, Shanghai 201306, China; zhiyahan@sdju.edu.cn (Z.H.); c2441693327@163.com (Y.C.); x2192792809@163.com (X.X.); 2 School of Intelligent Manufacturing and Control Engineering, Shanghai Polytechnic University, Shanghai 201209, China; 3 School of Material Science and Engineering, Tongji University, Shanghai 201804, China

**Keywords:** soft magnetic materials, nanocomposites, carbon nanotubes, high energy ball milling, Fe-CNT composites, microstructure, magnetic properties

## Abstract

Fe-CNT composites were synthesized via mechanical ball milling, incorporating varying amounts of carbon nanotubes (CNTs) into iron powder at concentrations of 1 wt%, 2 wt%, and 3 wt%. The impact of different CNT contents on the phase structure, microstructure, and magnetic properties of the composites was examined. Raman spectroscopy and X-ray diffraction (XRD) analyses revealed that despite some damage, CNTs retained a predominantly one-dimensional nanostructure post-ball milling. Moreover, an increase in CNT content led to a gradual rise in grain size and lattice strain of the iron powder, attributed to the formation of solid solutions and iron–carbon compounds. Scanning electron microscopy (SEM) observations demonstrated that the majority of CNTs were integrated within the iron matrix particles, with a minority either partially embedded or entirely unembedded on the iron powder surface. With higher CNT concentrations, local CNT agglomeration emerged and intensified. Vibrating sample magnetometer (VSM) measurements indicated that Fe-CNT composites exhibited enhanced saturation magnetization (2.25%) and reduced coercivity (91.74%) compared to pure iron, underscoring the potential of CNTs in enhancing the magnetic properties of iron powder.

## 1. Introduction

Due to their exceptional electrical, thermal, and mechanical properties, carbon nanotubes are utilized as reinforcement agents in metal matrix composites to improve their performance [1]. They have found extensive applications in various sectors including electronic components, aerospace, and national defense.

Carbon nanotube-doped metal matrix composites, including aluminum, magnesium, copper, titanium, silver, nickel–aluminum, nickel–iron, iron–cobalt, and iron, are of significant interest for high-strength and lightweight structural applications. Research in this area has primarily concentrated on process challenges, mechanical characteristics, and reinforcement mechanisms, leading to notable advancements [1,2,3,4]. For instance, incorporating CNTs into Al through high-energy ball milling and adding 15 vol% carbon nanotubes to a copper matrix enhances yield strength [5]. For the Cu-Ti alloy, it achieves a tensile yield strength of 515 MPa, tensile strength of 542 MPa, elastic modulus of 95.6 GPa, and elongation of 10.4%. In the iron–cobalt alloy, 0.5 vol% carbon nanotubes boost hardness by 18% (1 vol% graphene nanoplatelets) and achieve a maximum magnetic induction of 2.39 T [6]. Notably, research on carbon nanotube-reinforced iron-based composites is limited, primarily focusing on mechanical properties like hardness and strength [1,7,8,9,10,11], with minimal exploration of their magnetic characteristics [12,13,14,15,16]. Consequently, further investigations into the structure and magnetic properties of carbon nanotube-reinforced iron-based composites are warranted.

Numerous studies have demonstrated the crucial role of interfacial bonding quality between carbon nanotubes and metal matrices in determining composite properties [17,18,19]. High-energy ball milling is a widely recognized method for achieving homogeneous dispersion of carbon nanotubes within metal matrices, albeit at the expense of causing structural damage to the nanotubes, such as shortening and amorphization [20,21]. This structural damage triggers various interfacial reactions, including the formation of solid solutions or carbides, which significantly impact interfacial bonding strength. Notably, in the case of transition metals, there is potential for 3d-2p hybridization between their vacant 3d orbitals and the graphite structure of carbon nanotubes [22], facilitating favorable interfacial bonding [18,19]. The extent of structural damage and interfacial reactions during high-energy ball milling is intricately linked to the carbon nanotube content. Therefore, investigating the influence of carbon nanotube content on the structure and magnetic properties of iron-based carbon nanotube composites holds substantial importance.

In this study, Fe-CNT composites were synthesized via high-energy ball milling, followed by a comprehensive analysis of the impact of carbon nanotube concentration on the phase composition, morphology, and magnetic characteristics of the composites.

## 2. Materials and Methods

Water-atomized iron powder (particle size ≤ 150 μm, purity ≥ 99.5%, Tianjiu Metal Material Co., Ltd., Changsha, China) and carbon nanotubes (average outer diameter: 1–2 nm, length: 1–3 μm, purity ≥ 95%, Shanghai Aladdin Biochemical Technology Co., Ltd., Shanghai, China) were utilized as raw materials. The experimental procedure for fabricating Fe-CNT composites involved initially pre-mixing the iron powder and carbon nanotubes (at CNT contents of 1 wt%, 2 wt%, and 3 wt%, respectively) in a V-shaped powder mixer for 15 min. Subsequently, the mixture underwent dry-milling in a planetary ball mill (1-SL, Qingdao Union Machinery Co., Ltd., Qingdao, China) for 2 h. This milling process employed 304 stainless steel ball-milling jars and 304 stainless steel grinding balls, with a ball-to-powder ratio of 50:1, a rotation speed of 200 rpm, and the introduction of an argon protective atmosphere to prevent oxidation. The resulting ball-milled powder was then annealed at 600 °C for 2 h to relieve internal stress, with continuous maintenance of an argon protective atmosphere. Additionally, to assess the impact of carbon nanotubes on magnetic properties, pure iron powder without doping was prepared under identical conditions.

The soft magnetic powders were subjected to analysis of crystal structure and phase composition using an X-ray diffractometer (XRD) with Cu-Kα radiation, operating at 30 kV and 30 mA (DX-2007, Dandong fangyuan Co., Ltd., Dandong, China). Internal structural features of carbon nanotubes were characterized using a Raman spectrometer (Lab RAM HR-Evolution, HORIBA Scientific, Paris, France) with a laser wavelength of 532 nm, a spectral range of 200–210 nm, and a test range of 800–200 cm^−1^. The microstructure morphology of composite powders doped with carbon nanostructures was observed using a scanning electron microscope (SEM) (Nova NanoSEM 450, FEI, Portland, OR, USA). The hysteresis loops of the soft magnetic powders were measured using a vibrating sample magnetometer (VSM) (Model 7407, Lakeshore, Westerville, OH, USA) to analyze static magnetic properties including saturation magnetization, coercivity, and remanence.

## 3. Results

### 3.1. Phase Composition of the Fe-CNT Composites

**Figure 1:**
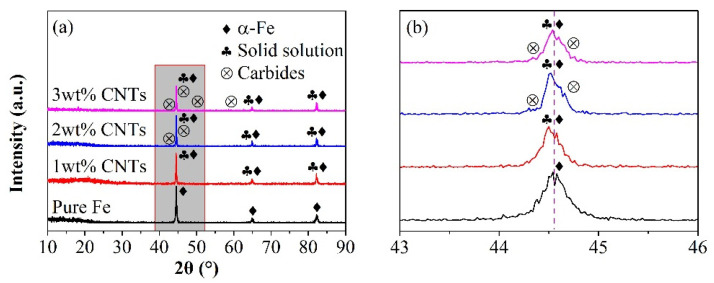
(**a**) XRD patterns of Fe-CNT composites with different CNTs contents; (**b**) partial enlarged image.

Figure 1a illustrates X-ray diffraction (XRD) patterns of Fe–carbon nanotube (CNT) composites with varying CNT contents (1 wt%, 2 wt%, 3 wt%). All samples display three prominent peaks at approximately 44.67° (110), 65.02° (200), and 82.33° (211), indicative of a typical body-centered cubic crystal structure in Fe-CNT primarily comprising the α-Fe phase. Relative to pure iron, the diffraction peaks of Fe-1 wt% CNTs exhibit a slight shift towards lower angles with reduced intensities, implying the formation of a solid solution identified as ferrite α-(Fe, C). With increasing CNT content, iron carbide phases emerge in the Fe-CNT composites, predominantly identified as Fe_3_C cementite. Comparison of the diffraction patterns reveals that, in contrast to Fe-1 wt% CNTs, the diffraction peaks of Fe-2 wt% CNTs and Fe-3 wt% CNTs shift towards higher angles, with the degree of shift escalating with rising carbon content, as depicted in the magnified XRD pattern excerpt in Figure 1b. Consequently, excessive CNT incorporation results in the precipitation of iron carbide phases like cementite at the iron–CNT interface, compromising the magnetic properties.

The lattice parameters of Fe-CNT composites exhibited an increase in comparison to pure iron, as detailed in Table 1. This phenomenon primarily arises from the creation of a solid solution through the incorporation of carbon atoms into the iron matrix during the ball milling process [23]. Nevertheless, a decline in lattice parameters was observed once the CNT content surpassed 1 wt%. This trend suggests that with higher CNT concentrations, carbon saturation within the iron matrix occurs, leading to the precipitation of iron–carbon compounds [12].

The grain size of the Fe-CNT composites was determined using the Scherrer formula applied to the XRD pattern [24]. Scherrer’s formula is as follows:

(1) $$D=\frac{k\lambda }{\beta \cos\theta }$$

where *D* is the grain size, *k* is the Scherrer’s constant, *β* is the FWHM and 2θ is the Bragg angle.

Table 1 illustrates that the grain size of the composite increased progressively with higher carbon nanotube content. Under deformation, fracture, and rewelding processes, the iron powder particles exhibited growth in a flattened orientation along the vertical axis. This behavior aligns with previous findings indicating a consistent upward trend in grain size [25,26].

The lattice strain in Fe-CNT composites is lower compared to pure iron powder. This reduction can be attributed to the formation of numerous small pores within the iron powder particles due to carbon nanotube aggregation, which can absorb some of the residual strain. Additionally, the nano-scale cavities in carbon nanotubes facilitate stress relaxation. However, an increase in CNT content leads to higher lattice strain, possibly due to localized agglomeration and uneven distribution of CNTs within the iron powder particles.

### 3.2. Structural Stability of CNTs

**Figure 2:**
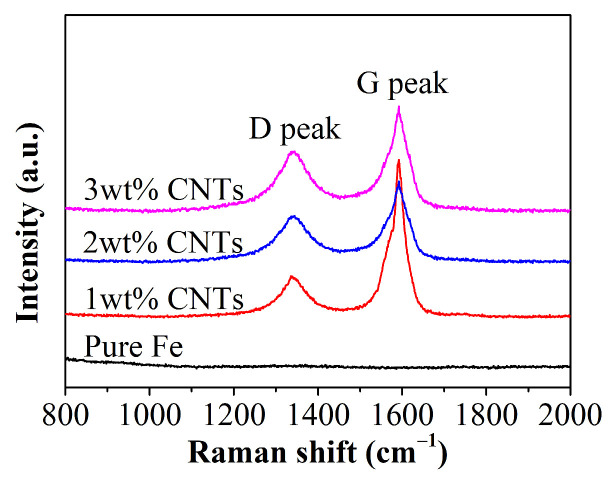
Raman spectrum of Fe-CNT composites.

Raman spectroscopy was utilized to assess the structural stability of carbon nanotubes (CNTs) by conducting tests and analyses on a composite powder of Fe-CNT, as depicted in Figure 2. The results reveal that all samples demonstrate two distinctive peak positions characteristic of CNTs: the D band at approximately 1344 cm^−1^ and the G band at around 1598 cm^−1^. Specifically, the G band is attributed to the graphite structure of sp^2^ hybridized carbon bonds, whereas the D band is associated with defects within the graphite structure.

Comparison of the Raman spectra of Fe-CNT composites reveals that with increasing carbon nanotube content, the G peak shifts to higher wavenumbers, exhibiting a shorter and broader profile. In contrast, the D peak experiences a slight shift to lower wavenumbers. These observations suggest that the ball-milling process induces damage to the graphite structure, leading to an increase in defect density [27,28].

The ratio of the intensity of the D peak to that of the G peak, denoted as (I_D_/I_G_), serves as a quantitative measure of defect density [10,27], as detailed in Table 2. In Fe-CNT composites, the (I_D_/I_G_) values are consistently low, indicating a higher defect density compared to pure CNTs while suggesting the preservation of the graphite structure [13]. With an increase in the carbon nanotube concentration, the (I_D_/I_G_) ratio gradually rises, likely attributed to the heightened presence of (sp^2^) hybridized carbon rings in high CNT content composites. Additionally, the elevated ratio may result from the cleavage of (sp^2^) carbon bonds, leading to the formation of minor quantities of amorphous carbon, solid solutions, and iron–carbon compounds [25,26,29].

### 3.3. Microstructure of the Fe-CNT Composites

**Figure 3:**
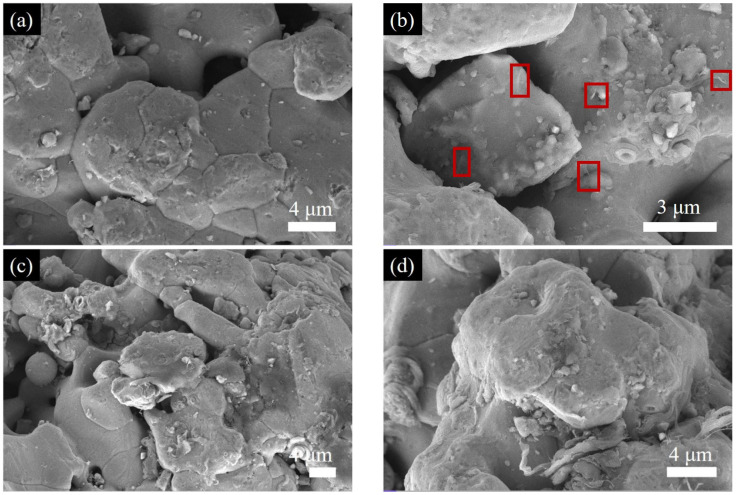
SEM image of Fe-CNT composite: (**a**) Pure Fe, (**b**) Fe-1 wt% CNTs, (**c**) Fe-2 wt% CNTs, (**d**) Fe-3 wt% CNTs.

The surface morphology of pure iron powder post-ball milling, devoid of attached CNTs, is depicted in Figure 3a. Figure 3b–d illustrate the morphological characteristics of Fe-CNT composites with varying carbon nanotube concentrations. A notable observation is the considerable reduction in CNT size across all samples compared to the pristine materials, with the majority measuring less than 1 μm. This reduction signifies that the high-energy ball milling process induces damage to the CNT length, leading to size diminution [30]. Despite the size reduction, the CNTs largely preserve their one-dimensional structure, as evidenced by Raman spectroscopy results.

Figure 3b–d illustrates that the majority of carbon nanotubes (CNTs) are integrated within the iron matrix metal particles, indicating potential robust interfacial bonding. Conversely, a minority of CNTs exhibit partial embedding or remain unembedded on the iron powder particles surface, consistent with prior literature [13]. This phenomenon primarily arises from the dispersion, fragmentation, refinement, cold welding, and alloying of powder particles occurring throughout the ball milling process [31].

Upon comparing and analyzing the dispersion of carbon nanotubes (CNTs) within Fe-CNT composites, it is evident that at a lower CNT concentration (1 wt%), CNTs exhibit a relatively homogeneous distribution within the iron matrix, as depicted in Figure 3a. As the CNT concentration increases to 2 wt%, agglomeration of CNTs initiates, as illustrated in Figure 3b. Subsequently, at 3 wt% CNT concentration, agglomeration becomes more pronounced, as demonstrated in Figure 3d. This localized agglomeration significantly undermines interfacial bonding, consequently impeding the overall performance of Fe-CNT composites.

### 3.4. Magnetic Properties of the Fe-CNT Composites

**Figure 4:**
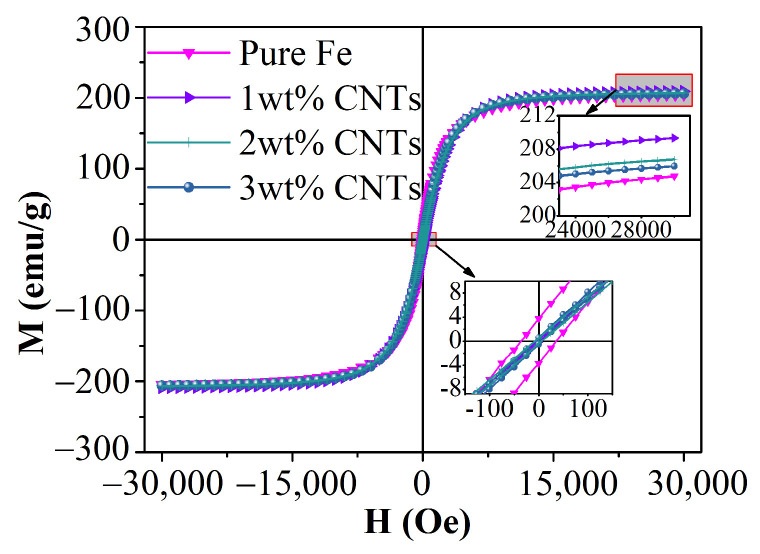
Magnetic hysteresis loop of Fe-CNT composites.

Figure 4 illustrates the hysteresis loops of the Fe-CNT composites, demonstrating consistent ferromagnetic characteristics across all samples. The findings reveal an enhanced saturation magnetization (M_s_) and a reduced coercivity (H_c_) in the Fe-CNT composites compared to pure iron powder, as evidenced by the detailed analysis provided in the figure.

As cited in References [32,33], ball milling typically induces strain accumulation within pure iron powder particles, leading to a decline in magnetic properties characterized by reduced saturation magnetization and increased coercivity. However, post-ball milling, the magnetization of Fe-CNT composites can increase by 2.25%, while coercivity can decrease by 91.74%, indicating a positive impact of carbon nanotubes on the magnetic properties of iron powder, as shown in Table 3. Carbon nanotubes exhibit ferromagnetic properties due to their preparation with iron as a catalyst [34]. This effect is primarily attributed to the interfacial interaction between carbon nanotubes and iron powder. Following ball milling treatment, carbon nanotubes can be effectively integrated into the iron powder matrix, establishing a robust interfacial bonding structure with high cohesion, ensuring strong bonding strength [12]. Then, the refinement effect of ball milling on composite powder particles plays a crucial role. Studies [12,13] have shown that when the particle size of the composite powder post-ball milling falls within the 35–40 nm range, the material typically adopts a single magnetic domain structure and interfaces coherently with carbon nanotubes. Under an external magnetic field, the magnetic domains of the composite material align neatly along the applied magnetic field, resulting in a notably high saturation magnetization. Furthermore, partial damage to carbon bonds in carbon nanotubes due to ball milling facilitates the precipitation of available carbon atoms at dislocations of adjacent iron crystals, promoting a 3d-2p hybridization reaction and forming a smooth CNTs-Fe matrix interface, positively influencing remanence. This enhanced remanence contributes to an overall improvement in saturation magnetization [12]. The coercivity of the composite powder is primarily influenced by factors such as grain size, dislocation defects, and stress. X-ray diffraction (XRD) analysis reveals that the powder with this doping level exhibits minimal internal stress, resulting in the least hindrance to magnetic domain movement and consequently lower coercivity.

**Figure 5:**
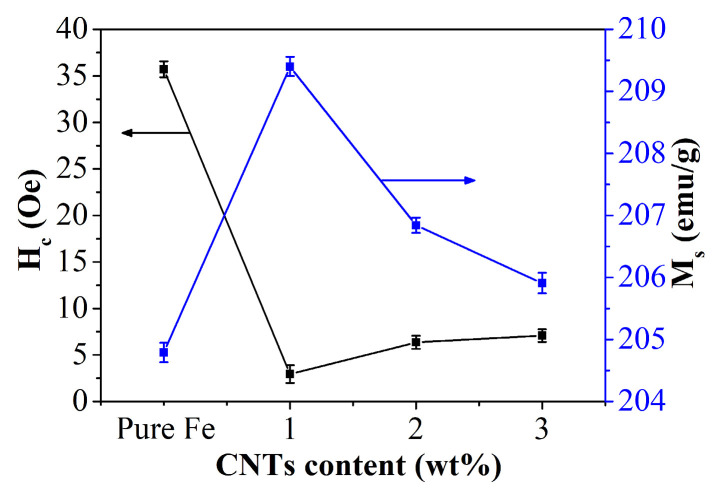
H_c_ and M_s_ of Fe-CNT composites.

Figure 5 illustrates the saturation magnetization (M_s_) and coercivity (H_c_) of Fe-CNT composites with varying amounts of carbon nanotube additions. The data reveals a non-linear relationship where M_s_ initially rises and then declines with increasing carbon nanotube content, while H_c_ exhibits the opposite trend. Specifically, at a carbon nanotube addition of 1 wt%, the composite demonstrates peak saturation magnetization and minimal coercivity. This behavior is predominantly attributed to the even dispersion of carbon nanotubes, as elucidated in the preceding SEM analysis.

## 4. Conclusions

The Fe-CNT composites were fabricated via mechanical ball milling, preserving the one-dimensional nanostructure of carbon nanotubes and ensuring their uniform dispersion within the iron matrix. With increasing CNT content, local agglomeration gradually emerges, causing some structural damage to the CNTs and leading to the formation of solid solutions and iron–carbon compounds within the iron matrix. The Fe-CNT composites exhibit enhanced M_s_ and reduced H_c_ compared to pure iron. This enhancement in M_s_ is primarily attributed to the strong interfacial interaction achieved through effective interfacial bonding and high cohesion between CNTs and iron powder post-ball milling. The composite powder displays reduced particle size, a single magnetic domain structure, and a coherent interface with CNTs, facilitating the alignment of magnetic domains along an external magnetic field and resulting in high M_s_. The partial damage to carbon bonds in CNTs during ball milling prompts the precipitation of available carbon atoms at dislocations of neighboring iron crystals, facilitating 3d-2p hybridization reactions and promoting a smooth CNTs-Fe matrix interface, thereby enhancing remanence and overall saturation magnetization. Additionally, CNTs synthesized using iron as a catalyst exhibit inherent ferromagnetism. The Hc is directly influenced by grain size, lattice strain, and material density. Among the samples, the Fe-1 wt% CNT composite demonstrates optimal CNT dispersion, exhibiting the highest M_s_ and the lowest H_c_.

## Figures and Tables

**Table 1:** Grain size, lattice parameters and lattice strain of Fe-CNT composite.

Results	Pure Fe	Fe-1 wt% CNTs	Fe-2 wt% CNTs	Fe-3 wt% CNTs
Lattice parameter (nm)	2.8739	2.8757	2.8748	2.8743
Grain size (nm)	33.1	39.6	69.5	88.6
Lattice strain (*ε* × 10^−3^)	3.3520	2.0478	2.3970	2.4930

**Table 2:** Raman spectra characteristics of Fe-CNT composites.

Sample	Raman Peak Shift (cm^−1^)		I_D_/I_G_
	D Band	G Band	
Pure Fe	-	-	-
Fe-1 wt% CNTs	1341.34	1590.47	0.3
Fe-2 wt% CNTs	1340.49	1592.18	0.49
Fe-3 wt% CNTs	1339.63	1592.60	0.85

**Table 3:** Magnetic properties of Fe-CNT composites with different CNT content.

Sample	M_s_ (emu/g)	H_c_ (Oe)
Pure Fe	~205	36
Fe-1 wt% CNTs	~210	3
Fe-2 wt% CNTs	~207	6
Fe-3 wt% CNTs	~206	7

## Data Availability

The original contributions presented in this study are included in the article. Further inquiries can be directed to the corresponding authors.
